# French Bulldog Diagnosed With a Suspected Brain Tumor Shortly After Treatment for Spinal Arachnoid Diverticulum

**DOI:** 10.1155/crve/8302806

**Published:** 2026-04-12

**Authors:** Yuya Nakamoto, Kyosuke Hidari, Mei Matsuo, Miwa Nakamoto

**Affiliations:** ^1^ Neuro Vets Animal Neurology Clinic, Kyoto, Japan; ^2^ Veterinary Medical Center, School of Veterinary Science, Osaka Metropolitan University, Osaka, Japan, osaka-cu.ac.jp

**Keywords:** brain tumor, canine, French bulldog, glioma, spinal disease

## Abstract

An 8‐year‐ and 8‐month‐old spayed female 8.8‐kg French bulldog presented with a 4‐month history of progressive deterioration, followed by paresis of both pelvic limbs. Orthopedic examination revealed no abnormalities. Neurological examination revealed no findings suggestive of brain dysfunction. However, abnormal findings, including impaired conscious proprioception in both pelvic limbs and hyperreflexia of patellar, cranial tibialis, and gastrocnemius reflexes, suggested a spinal segmental lesion in the T3–L3 region. The dog was diagnosed with a spinal arachnoid diverticulum in the T11–T12 vertebrae (Day 1) by magnetic resonance imaging (MRI) and underwent surgical treatment (dorsal laminectomy with dural marsupialization) on Day 27. The dog was presented to the referral hospital on a weekly basis and underwent neurological examinations at each visit. Initial neurological signs included persistent bilateral pelvic limb proprioceptive deficits; no other new neurological abnormalities were noted. Epileptic seizures occurred on Days 43 and 57. Brain MRI performed on Day 91 revealed findings suggestive of a glioma. The dog was administered prednisone (0.5 mg/kg SID, PO) and zonisamide (5 mg/kg BID, PO). Neurological abnormalities, such as circling and left forelimb ambulatory monoparesis, became more noticeable. The dog developed status epilepticus and died on Day 209. The owner said that the frequency of seizures was approximately twice a month and had not increased, and seizure duration did not appear to have lengthened until Day 209. In this case, no direct history or neurological abnormalities suggestive of a lesion in the forebrain region were noted until the onset of epileptic seizures; however, the glioma was diagnosed within 3 months of spinal arachnoid diverticulum diagnosis. Therefore, the brain tumor might have been detected if a brain MRI had been performed when spinal cord disease was initially diagnosed. This report indicates that simultaneous brain MRIs should be considered when performing MRI examinations for spinal cord diseases. However, this report describes a single case and lacks a histopathological evaluation for a definitive diagnosis of glioma, meningioma, or other types of brain tumors.

## 1. Introduction

Over the past decade, French bulldogs have become significantly more popular in Japan, with a 1.4‐fold increase in Japan Kennel Club registrations from 7075 in 2014 to 10,129 in 2024, making them the seventh most commonly registered breed in 2024 [1]. Notably, French bulldogs are overrepresented in neurological (brain and spinal cord) diseases, the most common of which are brain tumors, intervertebral disc herniation, and spinal arachnoid diverticulum [2–4]. Advances in diagnostic imaging have increased the possibility of accidentally detecting asymptomatic lesions in various organs. In human medicine, incidental findings have been reported in the lungs, breasts, ovaries, colon, adrenal glands, and brain [5]. In veterinary medicine, incidental findings have been reported in the head, neck, thorax, abdomen, forelimbs, and hindlimbs [6]. In this study, we present the case of a French bulldog diagnosed with a brain tumor within 3 months of spinal arachnoid diverticulum treatment.

## 2. Case Presentation

An 8.8‐kg spayed female French bulldog, aged 8 years and 8 months, was referred to the Neuro Vets Animal Neurology Clinic (Neuro Vets) for evaluation of the abnormal gait of the pelvic limbs, which did not improve within 4 months despite medication with a nonsteroidal anti‐inflammatory drug. Three days before referral, examinations performed by the attending clinician revealed no abnormalities in the complete blood count or serum biochemical analyses. However, vertebral anomalies were observed in the thoracic spine upon radiographic examination of the chest and abdomen of the animal.

**Figure 1:**
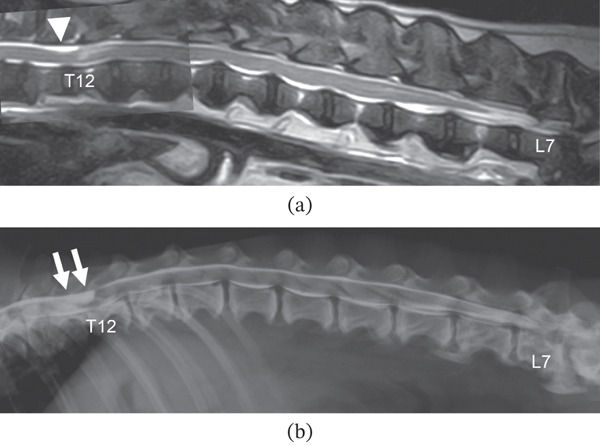
Magnetic resonance imaging (MRI) and myelography. (a) Sagittal plane T2‐weighted MRI showing a teardrop‐shaped fluid collection in the subarachnoid space of the T11–T12 vertebrae (arrowhead). (b) Left lateral myelography shows the injected contrast medium gradually dilating the subarachnoid space and ending abruptly as a teardrop‐shaped accumulation in the T11–T12 vertebrae (arrow). Based on these findings, a spinal arachnoid diverticulum was diagnosed.

At presentation to Neuro Vets (Day 1), a physical examination revealed the dog panting, with a body temperature of 39.8°C and a pulse rate of 132 beats/min. A neurological examination revealed that the patient′s mental status, behavior, posture, cranial nerves, sensation, and urinary function were normal. However, the pelvic limbs exhibited a paresis gait, decreased postural reactions, and hyperreflexia of the patellar, cranial tibialis, and gastrocnemius reflexes, indicating a lesion in the T3–L3 spinal cord segment. The clinical severity was classified as Grades 2–3 on the modified Frankel scale. Subsequently, magnetic resonance imaging (MRI) of the thoracolumbar spine was performed on the same day under general anesthesia with the dog in dorsal recumbent using a 0.4 Tesla MR imaging system with a permanent magnet (APERTO Lucent, Hitachi, Tokyo, Japan). Using a human knee coil, T2‐weighted (T2W) (FSE, TR/TE = 3500/120) and T1‐weighted (T1W) (SE, TR/TE = 300/13) images in the transverse and sagittal planes were obtained with and without 0.2‐mL/kg intravenous gadodiamide contrast (OMNISCAN, GE Healthcare Pharma, Tokyo, Japan). MRI revealed a teardrop‐shaped fluid collection in the subarachnoid space of the T11–T12 vertebrae, causing focal compression of the spine (Figure 1). On additional myelography, the injected contrast medium gradually dilated the subarachnoid space, eventually resulting in an abrupt teardrop‐shaped accumulation in the T11–T12 vertebrae.

Based on these findings, the dog was diagnosed with a spinal arachnoid diverticulum, for which the owner opted for surgical treatment. Until the surgical treatment, carprofen (Rimadyl, Zoetis Japan, Tokyo, Japan) was administered orally at a dose of 4.4 mg/kg every 24 h. On Day 27, dorsal laminectomy and dural marsupialization in the T11–T12 vertebrae were performed, and the dog was discharged from the Neuro Vets 5 days later (Day 32). As part of postoperative rehabilitation, massage of the muscles in both hind legs and bicycle pedaling exercises were performed. The follow‐ups occurred approximately weekly at the referring hospital; neurological examinations revealed an improvement in bilateral pelvic limb proprioception (Grade 2); no other significant abnormalities attributable to the spinal arachnoid diverticulum were identified. The dog was presented to the Neuro Vets for follow‐up of the spinal arachnoid diverticulum on Day 60. At that time, the owner complained of seizures on Days 43 and 57. Therefore, we suggested brain MRI and cerebrospinal fluid (CSF) analyses.

**Figure 2:**
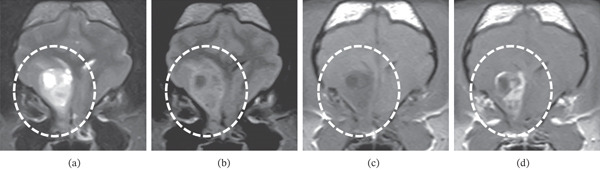
Magnetic resonance imaging findings. An intra‐axial–occupying lesion is observed in the right fronto‐olfactory cortex region (encircled), which is hyperintense on (a) T2‐weighted and (b) fluid‐attenuated inversion recovery images, mildly hypointense on the (c) T1‐weighted image, and heterogeneous on the (d) post‐contrast T1‐weighted image. The imaging findings suggested a high‐grade glioma.

Neurological examination performed on Day 91 detected only bilateral pelvic limb proprioceptive deficits (Grade 2). No abnormalities were observed in complete blood count or serum biochemical analyses. A 0.4‐T brain MRI was performed under general anesthesia with the patient in the prone position using a human wrist coil to obtain T2W (FSE, TR/TE = 3440/100), fluid‐attenuated inversion recovery (FLAIR; FIR, TR/TE = 8292/78), and T1W (SE, TR/TE = 400/15) images in the transverse, sagittal, and dorsal planes, with and without 0.2 mL/kg of intravenous gadodiamide contrast (OMNISCAN, GE Healthcare Pharma, Tokyo, Japan). MRI revealed an intra‐axial lesion in the right fronto‐olfactory cortex (Figure 2). The mass showed hyperintensity on T2W and FLAIR, mild hypointensity on T1W, and heterogeneous enhancement on postcontrast T1W images, with localized brain edema around the mass. Analyses of CSF samples collected from a cerebellomedullary cistern revealed a normal total cell count (1 WBC/*μ*L) and an increased protein concentration (68.5 mg/dL). Cytological examination of the CSF revealed scattered mononuclear cells. Based on these findings, the most likely diagnosis was a brain tumor such as a glioma. Among other options (resection, radiation therapy, and chemotherapy), due to financial constraints, the owner elected for symptomatic therapy, including prednisone (Prednisolone, Takeda Pharmaceutical Company, Tokyo, Japan) 0.5 mg/kg PO every 24 h and zonisamide (Consave, Bussan Animal Health Company, Osaka, Japan) 5 mg/kg PO every 12 h. The case progression was evaluated approximately every 3 weeks at the referring hospital, per the owner′s request. Evaluations included interviews with the owner regarding the dog′s behavior at home, seizure frequency, and seizure duration, along with neurological examinations conducted at the hospital. The doses of prednisone and zonisamide remained the same throughout the treatment period. The dog was able to lead a normal daily life; however, right‐sided circling and left forelimb ambulatory monoparesis were observed on Day 105, and a tendency to wander became apparent on Day 179. According to the owner′s interview, the frequency of seizures was approximately twice a month, and they did not feel that it was increasing. They also reported that the seizure duration did not appear to be lengthening. On Day 209, the patient developed status epilepticus and died calmly. An autopsy was not possible in this case.

### 3. Discussion

Brain tumors and spinal arachnoid diverticula are neurological disorders that commonly occur in French bulldogs [2, 3]. Gliomas account for a high proportion of brain tumors in French bulldogs. Histopathological examinations are required for a definitive diagnosis of glioma; classification as oligodendroglioma, astrocytoma, or undefined glioma; and further subclassification as low‐grade or high‐grade [7, 8]. However, a presumptive diagnosis can be made using MRI [2, 9]. MRI features commonly consistent with glioma include intra‐axial single lesions, heterogeneous, ovoid to amorphous, well‐defined to infiltrative, T2W isointense to hyperintense, and T1W isointense to hypointense mass lesions with variable contrast enhancement. Other findings were cyst‐like lesions, intralesional hemorrhage, vasogenic edema, and mass effects [9]. Particularly, ring‐like contrast enhancement is associated with malignancy. When contrast enhancement occurs, it often indicates a high‐grade malignancy [10]. In this study, the brain MRI findings of the dog were similar to those previously reported, and CSF analyses ruled out inflammatory disease; therefore, a diagnosis of glioma was considered appropriate, indicating a high‐grade malignancy.

The reported incidence of glioma in the fronto‐olfactory cortex ranges from 30% to 42% [9, 11]. The fronto‐olfactory cortex is called a silent area, and epileptic seizures are commonly the only clinical sign of a lesion in the fronto‐olfactory cortex [12]. According to a previous study, 31.3% of patients with fronto‐olfactory cortex glioma only had epileptic seizures [9]. A study of 91 dogs histologically diagnosed with glioma reported that epileptic seizures were the most common primary complaint (62%), with seizures being the sole clinical sign in 9% of these cases [11]. In French bulldogs, the causes of epileptic seizures include not only brain tumors but also idiopathic epilepsy and meningoencephalitis of unknown origin [2–4]. In the present case, the abnormal gait of the pelvic limbs was attributed to the spinal arachnoid diverticulum, as the gait improved (from Grade 2–3 to Grade 2) following surgery for the condition. Conversely, the epileptic seizures were considered an early sign of glioma in the fronto‐olfactory cortex.

Prognostic factors affecting glioma survival include tumor grade and site of origin [9, 13]. Although grading must be based on histopathological findings, using MRI features for the tumor grading of suspected gliomas has moderate accuracy [11, 14]. However, histopathological brain tumor grading could not be performed in the dog in this case report because an autopsy was not performed. Given that gliomas arising in the fronto‐olfactory cortex have a poor prognosis [11], the prognosis in this case was considered poor at the time of diagnosis. Early detection of brain tumors may help guide the owner’s treatment decisions and improve the dog′s quality of life.

Treatment options for gliomas include conservative treatment, surgery, radiation therapy, and chemotherapy. The median survival periods following conservative treatment for glioma are 26 days [11] and 61 days [9]. The median survival periods when surgical treatment alone is performed are 66 days [15] and 124 days [11]. The median survival with radiation therapy alone is 489 days for intensity‐modulated radiation therapy [16] and 349 days for stereotactic radiation therapy [17]. The median survival period for chemotherapy alone was 39 days when lomustine was used [18]. Although only one case has been reported, survival periods of 213 days with carmustine and 190 days with temozolomide have been documented [18]. If a brain MRI had been performed at the time of the initial spinal cord disease diagnosis, the brain tumor could have been diagnosed 90 days earlier in this case. The dog presented with epileptic seizures on Day 43 of the disease; therefore, at the time of the initial visit, the brain tumor could have been diagnosed. Had cost not been a limiting factor, earlier diagnosis might have permitted consideration of glioma‐specific therapies, which could have influenced the decision to pursue surgical treatment for the spinal arachnoid diverticulum.

To the best of our knowledge, no case report has described brain diseases occurring shortly after the diagnosis of spinal cord diseases. Brain diseases account for approximately 20% of neurological diseases reported in French bulldogs, of which approximately 37% are brain tumors [2]. With the widespread use of imaging equipment and recognition of the importance of brain checkups in human medicine, the incidental discovery of asymptomatic brain tumors has been reported [19–22]. Screening examinations such as thoracic radiography and abdominal ultrasonography in dogs presenting with neurological signs have identified incidental abnormal findings, thereby influencing treatment decisions [23, 24]. In veterinary medicine, one dog was diagnosed with an incidental glioma by brain MRI [9]. Therefore, when performing MRI examinations to diagnose spinal cord conditions in French bulldogs, a brain MRI may be worth considering.

This case report had several limitations, including the lack of histopathological examination and the fact that MRI examinations of the head and thoracolumbar region were not performed simultaneously. This study would have been more informative if it had been possible to differentiate between tumors other than gliomas and to classify the glioma types based on histopathological results. Nevertheless, we considered the mass to be a glioma with higher potential, based on MRI features. The final tumor classification, based on histopathological diagnosis, is a critical factor influencing prognosis. Future studies should investigate the frequency of incidental brain disease discovery in French bulldogs and assess therapeutic efficacy based on the final tumor type.

## Author Contributions

Conceived and designed the analysis: Y.N.; data collection: Y.N., K.H., M.M., and M.N.; contributed data/analysis tools: Y.N.; performed the analysis: Y.N.; wrote the paper: Y.N.

## Funding

No funding was received for this manuscript.

## Disclosure

All authors have read and approved the final version of the manuscript for publication.

## Consent

Informed consent was secured from the owner regarding the use of clinical data for research purposes.

## Conflicts of Interest

The authors declare no conflicts of interest.

## Data Availability

All data generated or analyzed during this study are included in this published article.
